# Identification of a nuclear localization signal in the *Plasmodium falciparum* CTP: phosphocholine cytidylyltransferase enzyme

**DOI:** 10.1038/s41598-020-76829-1

**Published:** 2020-11-12

**Authors:** Richard Izrael, Lívia Marton, Gergely N. Nagy, Hajnalka L. Pálinkás, Nóra Kucsma, Beáta G. Vértessy

**Affiliations:** 1 Institute of Enzymology, Research Centre for Natural Sciences, 1117 Budapest, Hungary; 2 Doctoral School of Multidisciplinary Medical Sciences, University of Szeged, 6720 Szeged, Hungary; 3 Department of Applied Biotechnology, Budapest University of Technology and Economics, 1111 Budapest, Hungary; 4 Division of Structural Biology, Wellcome Centre for Human Genetics, University of Oxford, Oxford, OX3 7BN UK

**Keywords:** Image processing, Protein translocation, Confocal microscopy, Malaria, Cell biology, Cellular imaging

## Abstract

The phospholipid biosynthesis of the malaria parasite, *Plasmodium falciparum* is a key process for its survival and its inhibition is a validated antimalarial therapeutic approach. The second and rate-limiting step of the de novo phosphatidylcholine biosynthesis is catalysed by CTP: phosphocholine cytidylyltransferase (*Pf*CCT), which has a key regulatory function within the pathway. Here, we investigate the functional impact of the key structural differences and their respective role in the structurally unique pseudo-heterodimer *Pf*CCT protein in a heterologous cellular context using the thermosensitive CCT-mutant CHO-MT58 cell line. We found that a *Plasmodium-*specific lysine-rich insertion within the catalytic domain of *Pf*CCT acts as a nuclear localization signal and its deletion decreases the nuclear propensity of the protein in the model cell line. We further showed that the putative membrane-binding domain also affected the nuclear localization of the protein. Moreover, activation of phosphatidylcholine biosynthesis by phospholipase C treatment induces the partial nuclear-to-cytoplasmic translocation of *Pf*CCT. We additionally investigated the cellular function of several *Pf*CCT truncated constructs in a CHO-MT58 based rescue assay. In absence of the endogenous CCT activity we observed that truncated constructs lacking the lysine-rich insertion, or the membrane-binding domain provided similar cell survival ratio as the full length *Pf*CCT protein.

## Introduction

Malaria is a major global threat with around 50% of the world population being in danger of getting infected. The disease is caused by the parasites of the *Plasmodium* genus, most notably *Plasmodium falciparum.* Despite the immense effort to eradicate malaria worldwide, ca. 228 million cases of malaria have been reported in 2018 among which 405.000 cases were lethal^1^. The occurrence of drug-resistant parasite strains makes it even more difficult to eliminate the threat^2^. Therefore, the development of new compounds with different mechanisms of action is essential for effective therapy^3^. Currently, the artemisinin-based combination therapy (ACT) is the first-line of defence in many regions, although there are already parasite strains with delayed parasite clearance in many regions^4^. Essential biosynthetic pathways are in the focus of research to exploit the fundamental need of the *Plasmodium* parasite on processes such as DNA replication, nucleotide and phospholipid biosynthesis^5,6^.

During the intraerythrocytic life cycle, *Plasmodium* species produce up to 20 daughter parasite cells that egress from a single infected red blood cell in just 48 h^6^. This fast proliferation requires an excessive amount of membrane to be generated de novo. Therefore, the synthesis of phospholipid components is a process of key importance for the parasites, especially in the trophozoite and schizont stages. In eukaryotic cells, phosphatidylcholine (PC) is the major phospholipid component in most of the membrane structures^7^. Within the *Plasmodium* genus, PC is primarily generated in a three-step biosynthetic pathway called the Kennedy-pathway and that is partially supplemented by the methylation pathways of phosphoethanolamine and phosphatidylethanolamine^8^. Albitiazolium, a lead compound targeting the parasite PC biosynthesis, has already been successfully evaluated in human phase I and II clinical trials^9^, yet later in pediatric tests its high metabolic clearance was observed in children, which ultimately stopped its clinical development^10^. Nevertheless, the detailed analysis of the mode of action and pharmacokinetics of this drug could be utilized in future developments^10^.

**Figure 1:**
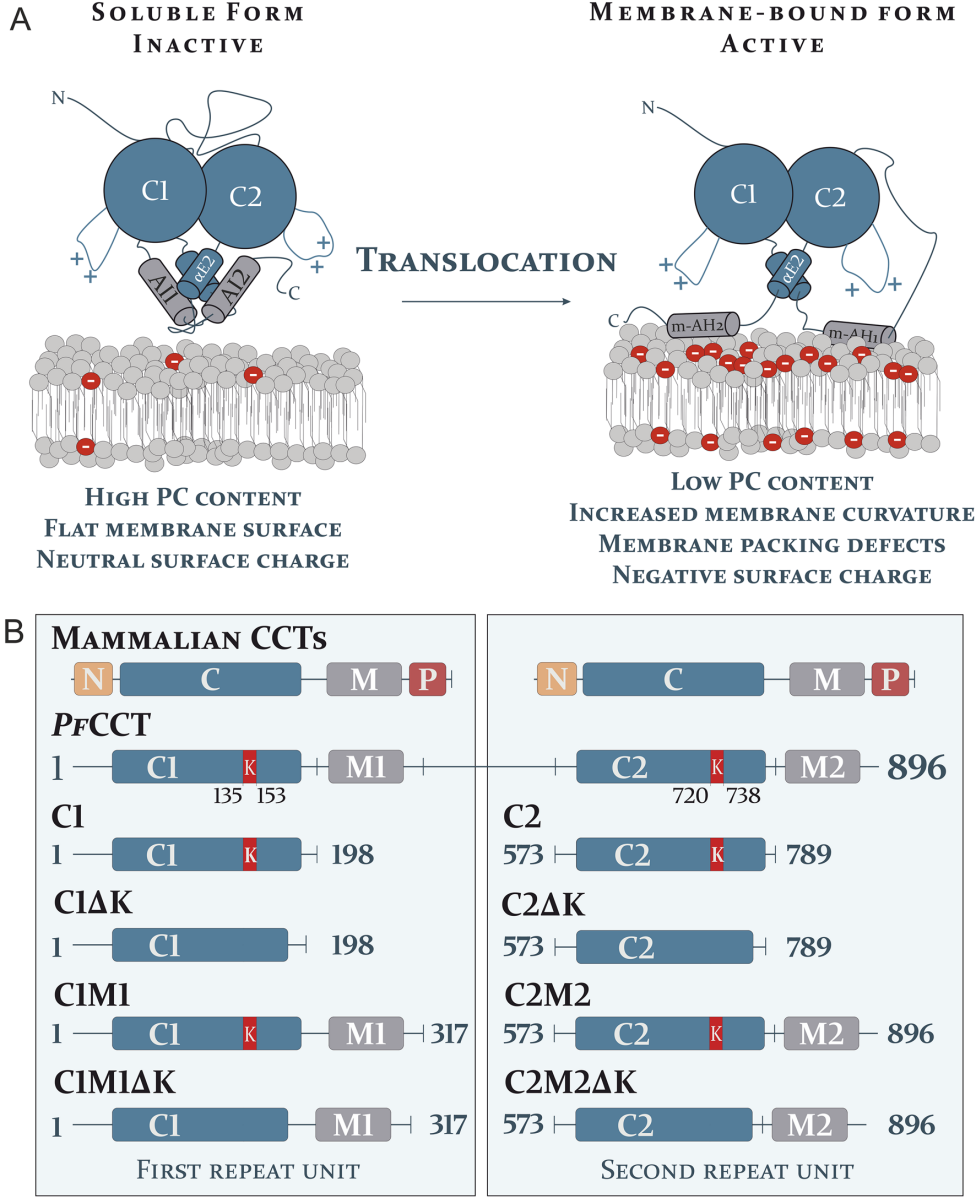
Molecular architecture and regulation of the CCT protein. (**A**) The conceptual regulatory mechanism of CCT on the macromolecular level. In the inactive state, an autoinhibitory (AI) helix in the membrane-binding (M—grey) domain inhibits the catalytic domain (C—blue) in the inactive state (left). The decreased PC content alters the physicochemical properties of membranes. This induces a conformational change in the M domain, turning the AI helix into the membrane-induced amphipathic helix (m-AH) that docks into the PC-depleted membrane surface and thereby releases the inhibition (right)^16^. (**B**) The schematic representation of mammalian CCT proteins and the truncated protein constructs of *Pf*CCT used in this study. N, C, M and P represent the N-terminal cap region, the catalytic domain, the membrane-binding domain and the region of phosphorylation, respectively. C1 and C2 constructs solely contain the catalytic domain of the first and second repeat unit of *Pf*CCT, respectively. C1M1 and C2M2 also include the respective putative membrane binding domains of each repeat unit. ΔK constructs lack the respective lysine-rich loops (red segments noted as K).

The second and rate-limiting step of the Kennedy-pathway is catalysed by the enzyme CTP:phosphocholine cytidylyltransferase (CCT), which converts the precursor phosphocholine (PhoC) to cytidine-5′-diphosphocholine (CDPCho) by incorporating CTP and releasing pyrophosphate as a by-product. CCT proteins of *Plasmodium* species, including the *Plasmodium falciparum* CCT (*Pf*CCT) are unique orthologues in terms of their architecture. While CCT enzymes regularly form a homodimer structure^11^, *Pf*CCT is a pseudo-heterodimer that contains both monomer units as two repeat units within a single polypeptide chain as a result of a gene duplication event^12^. The pseudo-monomer repeat units correspond to the basic domain structure of CCT enzymes, including the catalytic (C) domain and the putative regulatory membrane-binding (M) domain (Fig. 1A)^13^. However, other regions such as the N-terminal nuclear localization signal (NLS) and the C-terminal phosphorylation site present in mammalian orthologs have not been identified in *Pf*CCT (Fig. 1B)^12,14^. Within the active sites, the repeat units also contain the CTP-binding motif HxGH as well as the RTEG(I/V)ST(S/T) motif that overlaps with the N-terminus of the αE helix. The latter motif serves as a bridge between the C and M domains to assist membrane-binding induced regulatory changes^15^. Between the two pseudo-monomers (C1M1 and C2M2), a more than 200 amino acid long disordered inter-linker region is present, whose purpose has not yet been characterized (Fig. 1B). The precise molecular background of the *Pf*CCT regulatory mechanism has not been revealed in detail, but it is expected to be reminiscent to the mode of action described for the mammalian CCTs^16,17^. In the inactive soluble form, the catalytic domain of the CCT is inhibited by an autoinhibitory (AI) helix in the M domain^18,19^. PC-depleted membranes have altered physicochemical properties, which leads to increased negative surface charge and membrane curvature, creating membrane packing defects^17,20,21^. The CCT protein is able to sense these alterations and the M domain undergoes significant conformational changes. Subsequently, the AI helix releases the catalytic domain and turns into a membrane-induced amphipathic helix (m-AH) that docks into the PC-depleted membrane surfaces^15,21,22^. Intriguingly, in *Pf*CCT, the single continuous m-AH in mammalian CCTs^23^ is substituted by two separate amphipathic helices in the putative membrane binding domain, a shorter N-terminal and a longer C-terminal helices (m-AH-N and m-AH-C, respectively)^24^*.* Upon membrane tethering, the catalytic domain is released from autoinhibition, resulting in a dramatic increase of enzyme activity in mammalian CCTs^15,25^. Accordingly, an engineered CCT construct comprising the catalytic but not the membrane-binding domain possesses constitutive, lipid-independent enzyme activity^26^. Nevertheless, compared to its orthologs, *Pf*CCT has a moderate enzyme activity in the absence of lipid activators and it only shows a sixfold increase in activity in the presence of lipid vesicles^27^. The reason and the purpose of this difference compared to other orthologous CCTs is yet to be explored.

Another major difference between *Plasmodial* and mammalian CCTs concerns the number of enzyme isoforms. The vast majority of CCT proteins in higher eukaryotic organisms have two major isoforms, CCTα/CCT1 and CCTβ/CCT2, which localize in different cellular compartments and translocate to different membrane surfaces^28,29^. The dominant CCTα/CCT1 isoform is a nuclear protein that translocate to the inner nuclear membrane^22,30,31^, while CCTβ/CCT2 is a cytosolic isoform that primarily docks into the surface of the endoplasmic reticulum in the cytoplasm^32–34^. Interestingly, in cells with excessive need for PC synthesis e.g. pulmonary epithelial cells, CCTα can relocate to the cytoplasm, supposedly to enhance the PC biosynthetic process^35^. Furthermore, CCTα is able to shuttle between the nucleus and cytoplasm upon changes in the cellular PC homeostasis^33,36^. External stimuli such as phospholipase treatment^28^, addition of lipid activators (e.g. farnesol, oleate)^36,37^ or choline deprivation^38^, can be used in experimental systems to modulate CCT function and investigate its subcellular behaviour. In *Drosophila* S2 cells, CCT1 was able to relocate from the nucleus to the surface of cytoplasmic lipid droplets, but not in the mammalian 3T3-L1 cell line and differentiating human preadipocytes^22,37^. In the lower eukaryotic organism, *Saccharomyces cerevisiae* the sole CCT isoform is found in the nucleus^39,40^. However, in the rodent malaria parasite, *Plasmodium berghei,* the only CCT was found to be diffusely localized in the cell, both in the nucleus and cytoplasm^8^. *Plasmodium falciparum* also possesses only one CCT isoform, which localization is currently only known in schizont stages and was shown to be mostly diffuse in the parasite^41^.

In the present study, we investigate the functional impact of individual *Pf*CCT structural elements on subcellular localization and activity with several truncated constructs expressed in the CCT-mutant CHO-MT58 cell line (Fig. 1B). We found that the elimination of either the *Plasmodium-*specific lysine-rich insertion within the catalytic domain or the putative membrane-binding domain disrupted the nuclear localization of the protein. Furthermore, treatment of the cells with a translocation inducing enzyme, phospholipase C (PLC) resulted in a partial nuclear-to-cytosolic relocation of the full length *Pf*CCT and the second repeat unit construct. Previously, we started to characterize *Pf*CCT in a cellular context by its transient heterologous expression in the thermosensitive CCT-mutant CHO-MT58 cell line^42^. In this system, we have already demonstrated that *Pf*CCT is able to rescue the CHO-MT58 cells from apoptosis at the restrictive temperature^42^. Here, we performed rescue experiments with a set of truncated *Pf*CCT constructs encompassing the catalytic domain of the first or second repeat unit with optional presence of the local lysine-rich insertion and/or the adjacent membrane binding domain. All the truncated constructs retained their in vivo functionality by managing to rescue the cells with similar potential as the full length *Pf*CCT protein. Our results thus indicate that the sole presence of the catalytic domain is enough to rescue the cells at the restrictive temperature.

## Results

### Identification of a potential nuclear localization signal in *Pf*CCT

**Figure 2:**
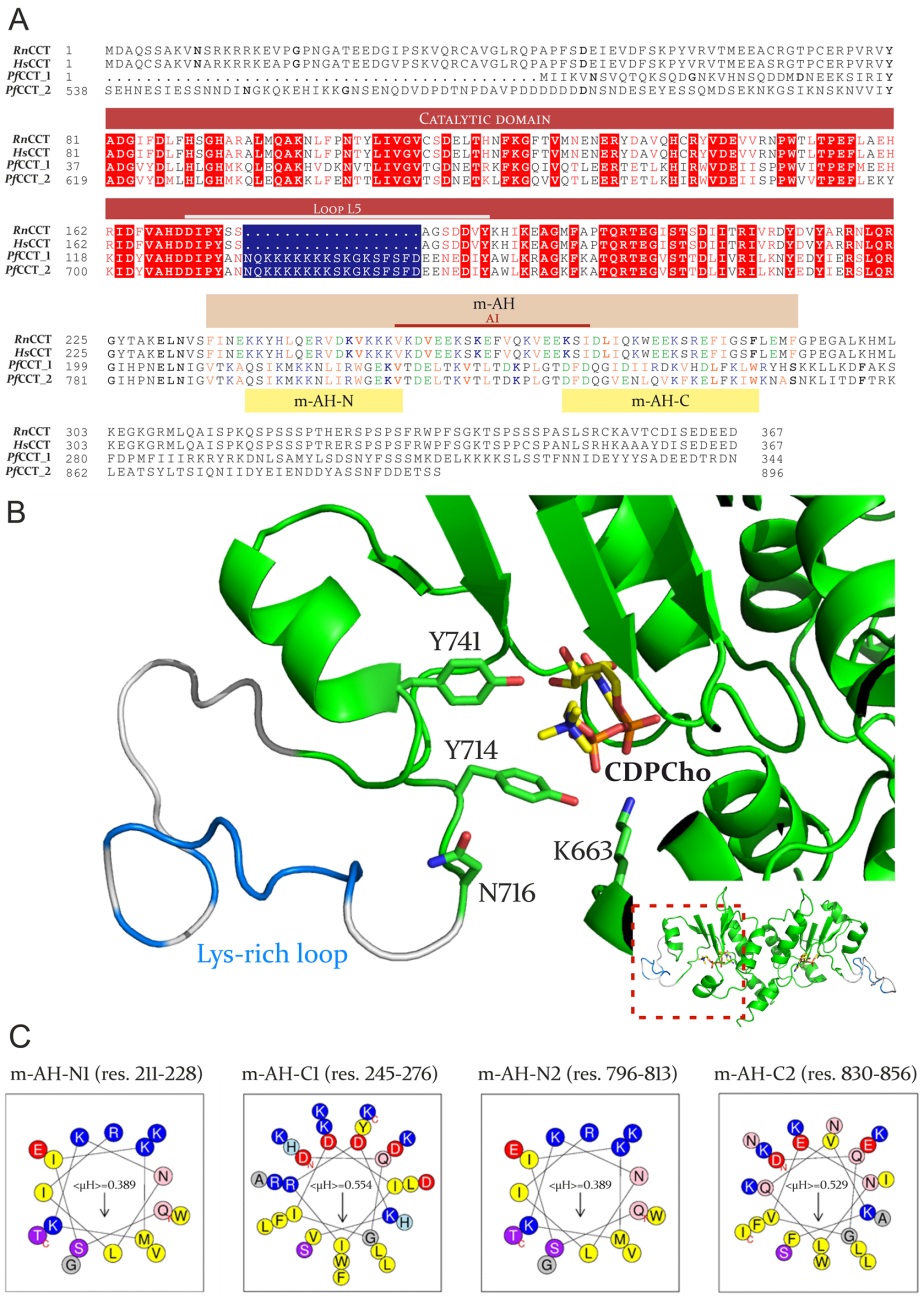
In silico analysis of PfCCT-specific protein segments. (**A**) The protein sequence alignment of the rat (*Rn*CCT), human (*Hs*CCT) and the two CCT repeat units of *P. falciparum* CCT (*Pf*CCT_1 and *Pf*CCT_2). Red boxes and red letters indicate the identical and similar amino acid residues within the catalytic domain, respectively. Blue box shows the *Plasmodium*-specific lysine-rich loop. Green, blue and yellow letters represent acidic, basic and hydrophobic residues in the membrane binding domain, respectively. The highly conserved catalytic domain is highlighted above the sequences (red) and a white line indicates the position of L5 loop. The membrane-induced amphipathic helix of *Rn*CCT (brown box) with the determined autoinhibitory helix (red line) is shown for reference. The two, previously hypothesized N- and C-terminal membrane-induced amphipathic helix of *Pf*CCT M domain is highlighted under the sequence with yellow boxes^24^. Alignment was generated with ESpript 3.0^45^. (**B**) Homology model of the active site of *Pf*CCT with one potential representative conformation of the flexible lysine-rich loop in a close-up view. Catalytically important residues in the proximity of L5 loop are highlighted, based on the crystal structure of *Pf*CCT (green, PDB: 4ZCS). The position of CDPCho (yellow) is shown in the active site. Blue colored line shows the position of the lysine residues on the main chain of the L5 loop (grey). (**C**) Helical representation of the putative membrane-induced amphipathic helices in the membrane-binding domain of the first and second repeat unit, respectively were made with HeliQuest^46^. <µH> represents the hydrophobic momentum of each helix. The abundance of basic residues (blue) supports the higher affinity of membrane-binding towards negatively charged membrane surfaces, whilst a few acidic (red) and the vast majority of hydrophobic residues (grey) found in the longer m-AH-C helices supposedly have a role in the detection of local H^+^ accumulation and support the docking into the membrane leaflets^47^.

The sequence alignment of *Pf*CCT with two mammalian orthologs, rat CCT (*Rattus norvegicus, Rn*CCT*)* and human CCT (*Homo sapiens, Hs*CCT) shows a highly conserved catalytic domain with one major exception (Fig. 2A). An 18 amino acid-long, lysine-rich insertion is present in the catalytically important L5 loop in both the first and second catalytic domains (C1 and C2) of *Pf*CCT, respectively^11^. The L5 loop is responsible for the coordination of the quaternary ammonium moiety of choline with residues Y131/Y714, N133/N716 and Y158/Y741 in C1/C2 and contributes to the formation of the composite aromatic box cleft^43,44^. We generated a homology model of *Pf*CCT to complete the recently resolved crystal structure (PDB: 4ZCS, Fig. 2B)^44^ by visualizing the 720–737 Lys-rich insertion that was deleted from the crystallized construct. The insertion appears as a highly flexible region, characterized by low QMEAN local quality scores with an average of 0.4. Therefore, the homology model shows only a representative position of this dynamic region. Notably, the deletion of the lysine-rich segment had no significant impact on the in vitro constitutive enzyme activity of a catalytic domain construct PfCCT_528–795_^12^.

However, other potential functions might be related to this segment. Firstly, the complex regulatory mechanism of CCT enzymes include several dynamic conformational changes, which could be altered by the lysine-rich loop. In addition, its presence may provide an explanation to the unique diffuse cellular localization of *Pf*CCT. As the large molecular weight (~ 105 kDa) of *Pf*CCT excludes the possibility of its passive nuclear transport, we hypothesize that the presence of a nuclear localization signal (NLS) in the protein is indispensable for nuclear accumulation. *In silico* predictions with *cNLSmapper* showed the possibility of a bipartite NLS in the lysine-rich loop with a score of 5.8, that indicates a weak NLS with the potential to partially relocate the protein to the nucleus. Based on its amino acid composition as well as the homology model of the *Pf*CCT protein, the lysine-rich loop is expected to be disordered and solvent-accessible region, that facilitates its recognition by *Plasmodial* nuclear import proteins. Since the nuclear import machinery must be able to access the signal peptide to effectively carry its cargo into the nucleus, the lysine-rich loop can be valid candidate to have an NLS function. Additionally, the Lys-rich insert might be associate with negatively charged membrane surfaces, given its pronounced basic character.

Two additional NLS predictions were found between residues 264–293 and 823–851 with a score 5.7 and 5.2, respectively. These regions both overlap with the presumed C-terminal peptide of the membrane-induced amphipathic helix of the M domain (m-AH-C, Fig. 2C). It was previously reported that the M domain is required for the shuttle between the nucleus and the cytoplasm^36^, therefore we attempted to analyse the effect of the truncation of the M-domain on the localization of *Pf*CCT.

**Figure 3:**
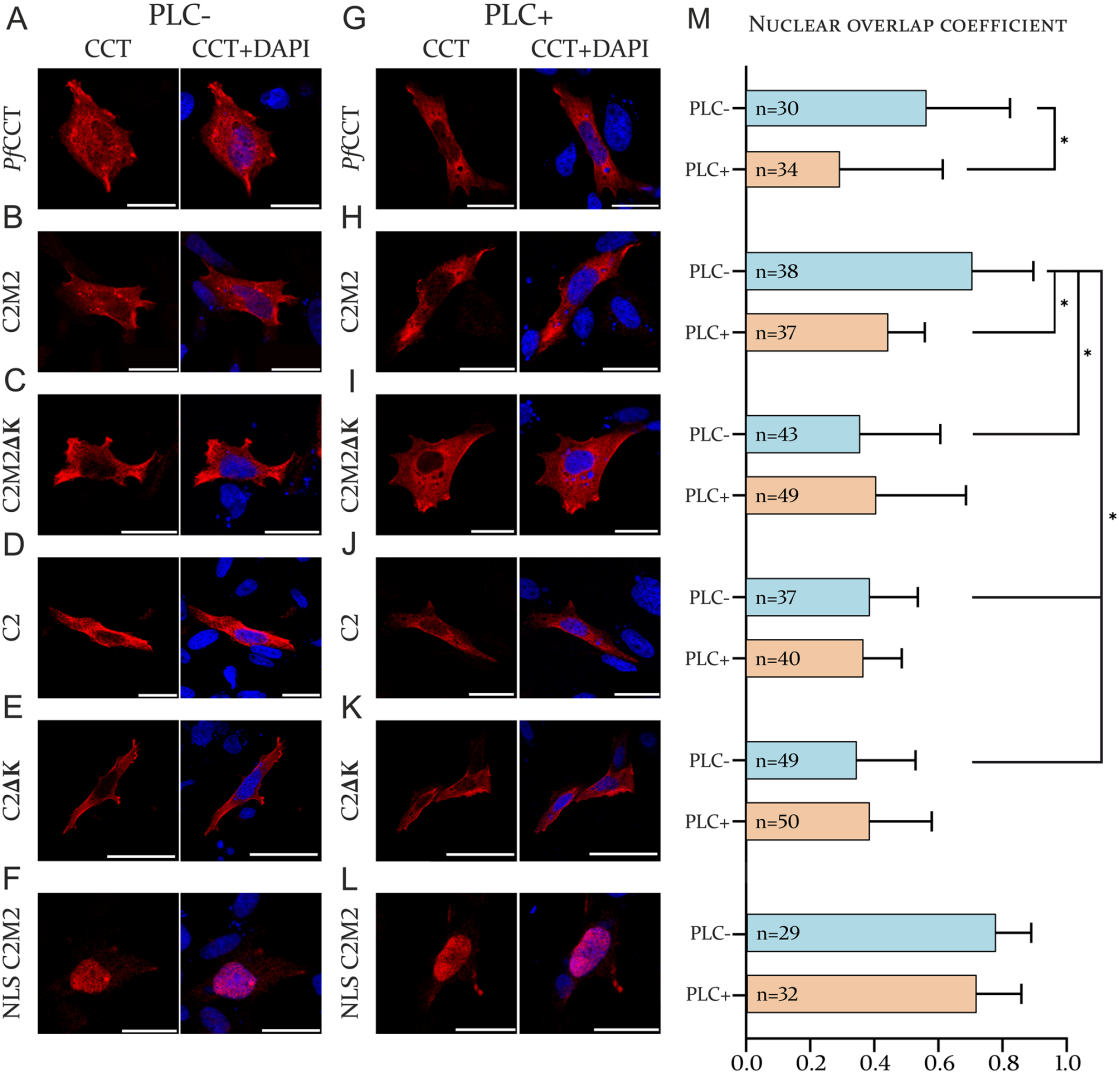
Colocalization analysis of *Pf*CCT construct in CHO-MT58 cells. Confocal immunofluorescence images of cells transfected with (**A**) the full length *Pf*CCT, (**B**) the second repeat unit C2M2 construct, (**C**) the lysine-rich loop truncated C2M2ΔK construct, (**D**) the membrane-binding domain truncated C2 construct, (**E**) C2ΔK and (**F**) SV40 NLS-tagged C2M2 construct. (**G**–**L**) Confocal images of cells transfected with *Pf*CCT constructs and treated with PLC. White lines indicate 50 µm scalebars. (**K**) Nuclear overlap coefficients were calculated with *Fiji*. The number of cells used for the calculation are indicated in the columns. *p < 0.001.

To elucidate the impact of the proposed regions on the nuclear localization, confocal microscopy-based colocalization analysis was applied. Both the full length *Pf*CCT and the second repeat unit construct, C2M2 has a mixed nuclear-cytoplasmic localization in CHO-MT58 cells (Fig. 3A,B). However, the lysine-rich loop truncated C2M2ΔK construct showed a predominantly cytosolic localization, which indicates the nuclear localization signal role of this insertion (Fig. 3C). Furthermore, the membrane-binding domain truncated second repeat unit construct, C2 also showed a decreased nuclear localization compared to the C2M2 construct and the full length *Pf*CCT protein (Fig. 3D,H,M). We also investigated the consequences of a double truncated C2ΔK construct, which showed a decreased nuclear accumulation similar to the C2 and C2M2ΔK constructs (Fig. 3E,K).

We wanted to further investigate the role of these segments on the translocation mechanism. For this, we used phospholipase C (PLC), an enzyme that hydrolyses PC in the cellular membrane structures and therefore simulates a PC-depleted membrane status in the cells^48,49^. The subcellular localization of the full length *Pf*CCT showed a peculiar pattern upon treatment, as the protein distribution became predominantly cytosolic (Fig. 3G). We observed the same change in distribution in case of the C2M2 construct (Fig. 3H). To test whether the relocation could be associated with the nuclear localization signal of *Pf*CCT, we created a simian virus 40 (SV40) NLS-tagged C2M2 construct as a positive control. Interestingly, this construct did not relocate to the cytosol which indicates that a stronger NLS keeps the protein in the nucleus even after PC depletion (Fig. 3F,L). We did not observe any changes in the localization of the C2M2ΔK, C2 and C2ΔK constructs upon PLC treatment (Fig. 3I–K).

### Functional impact of the lysine-rich loop on the rescue potential

**Figure 4:**
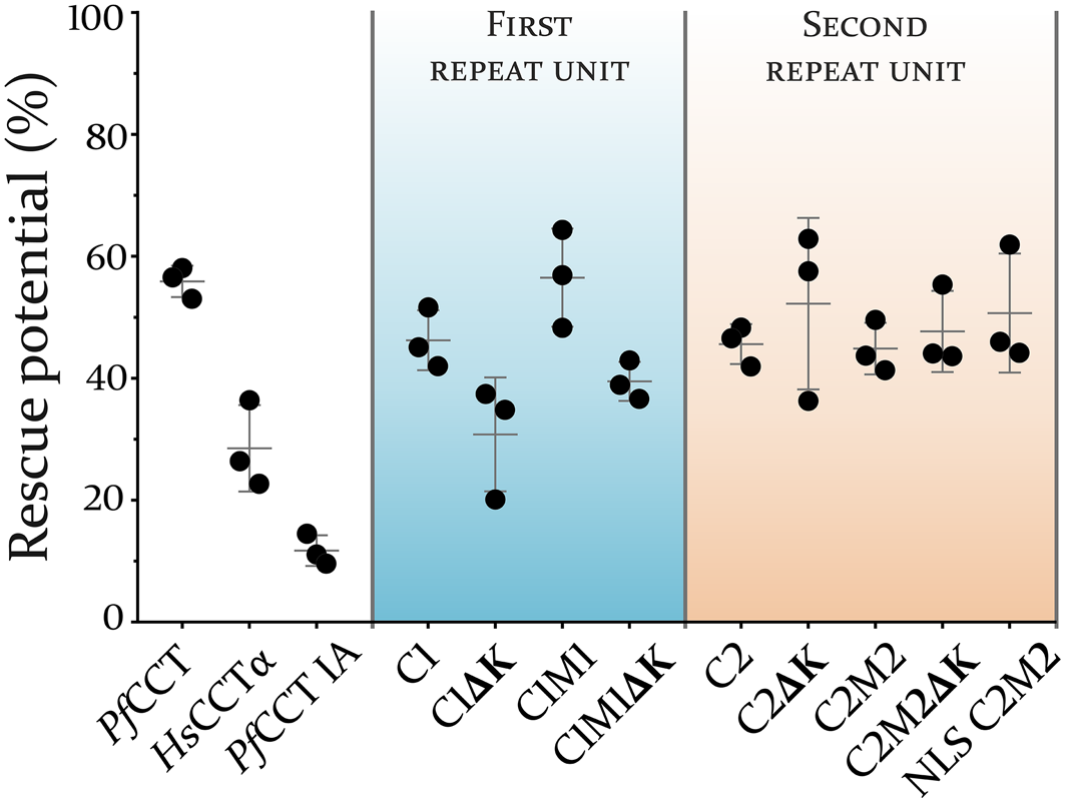
Rescue potential of the different *Pf*CCT constructs carried out in the thermosensitive CCT-mutant CHO-MT58 cell line system. Rescue potentials were determined as the quotient of surviving cells at 40 °C and 37 °C. Our results indicate that the sole presence of the catalytic domain is enough to rescue the cells at the restrictive temperature. All experiments were carried out in triplicates with averages ± S.D. being indicated. One-way ANOVA reveals no significant difference amongst the full length *Pf*CCT and the truncated constructs (p > 0.05). All constructs are significantly higher than our inactive *Pf*CCT mutant control (p < 0.01).

To evaluate the functionality of the different constructs in a cellular environment, we exploited the thermosensitive nature of the CCT-mutant CHO-MT58 cell line. Notably, these cells behave similarly to the parental CHO-K1 cell line at 37 °C, however, they are not viable at 40 °C, mainly due to thermal destabilizing effect of the CCT mutation^50,51^. Transient transfection of CHO-MT58 cells with the full length *Pf*CCT protein was shown to be able to rescue the cells with a rescue potential of 55.9 ± 2.6% (Fig. 4), which is in good correspondence with the data reported^42^. We also used the inactive (IA) mutant *Pf*CCT H45N H630N designed previously^42^ as a negative control that has a rescue potential of 11.7 ± 2.5%. In case of the constructs from the first repeat unit, C1M1 and C1 showed no significant difference in terms of cell survival, with a respective rescue potential of 46.2 ± 4.9% and 52.2 ± 10.9%. The second repeat constructs, C2M2 and C2 have similar results with a rescue potential of 45.6 ± 3.3% and 44.9 ± 7.5%, respectively. This suggests that the truncation of the putative M domain had no significant impact on the in vivo functionality of either the first or the second pseudo-monomer of the *Pf*CCT protein. The deletion of lysine-rich loop also did not perturb the activity as reflected in a rescue potential of 52.2 ± 10.9% and 47.7 ± 6.7% for C1M1ΔK and C2M2ΔK is, respectively. Remarkably, the double truncated constructs C1ΔK and C2ΔK displayed a difference regarding their activity. While the second repeat unit construct C2ΔK had a rescue potential of 52.2 ± 14.1%, the first repeat unit construct C1ΔK had around half this value, 30.8 ± 9.3. Furthermore, the mammalian CCT control *Homo sapiens* CCTα (*Hs*CCTα) was also found to be less potent in rescuing the cells as reflected by its rescue potential of 28.5 ± 7.1. While the underlying reason for these different rescue efficiencies remains unclear, notably, the rescue potential of all constructs tested is significantly (p < 0.01) higher than the inactive control *Pf*CCT H45N H630N, indicating CCT enzyme activity dependent rescue. We hypothesize this variability is due to the different transfection efficiencies of constructs in this study.

## Discussion

**Figure 5:**
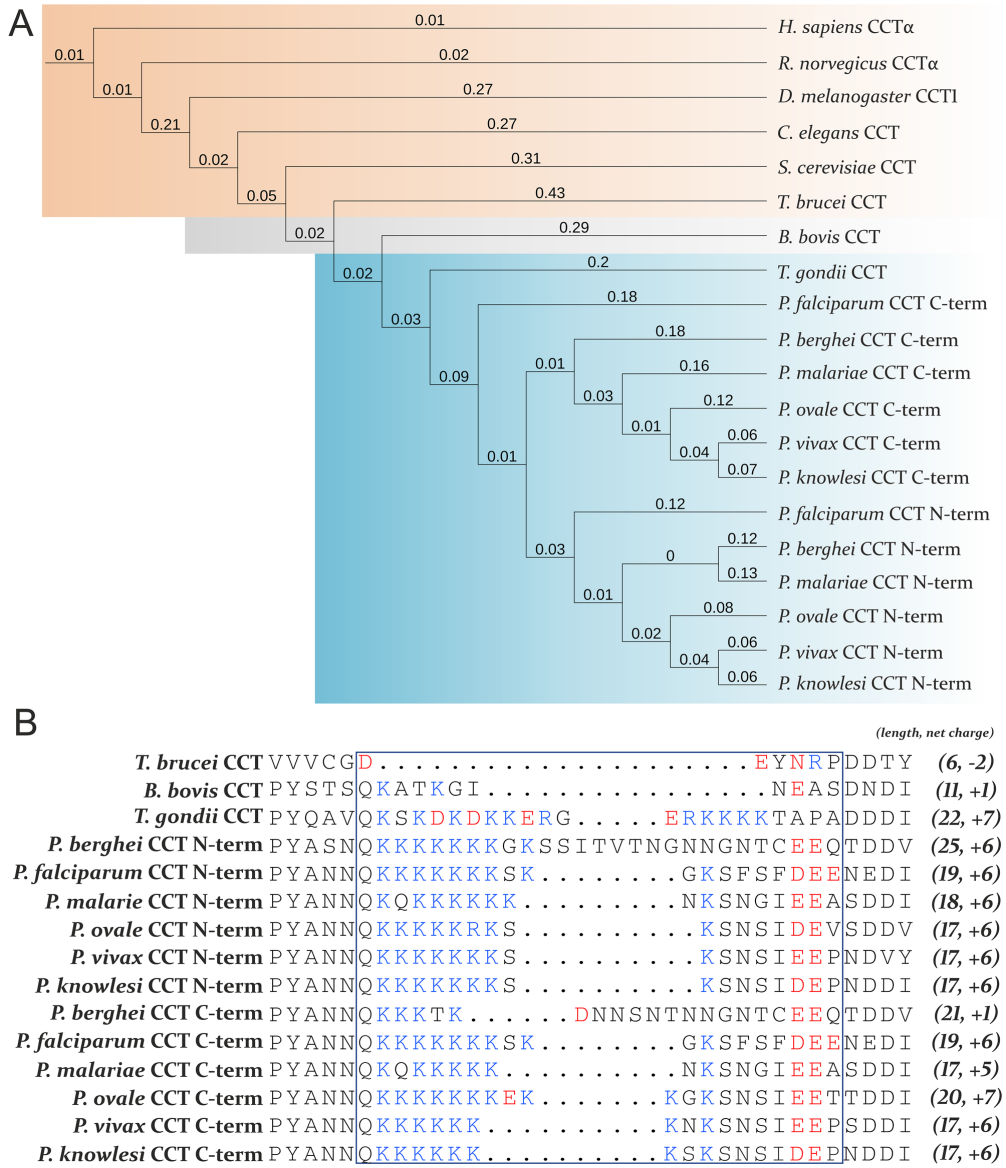
Alignment of the lysine-rich loop from protists and *Plasmodium* species infecting mammals. (**A**) Phylogenetic tree of the CCT enzymes were put into context regarding the presence of an insertion in the catalytic domain. Orange background indicates the lack of insertion in higher eukaryotic organisms and *Trypanosoma brucei*. *Babesia bovis* evolved in a similar direction and has a smaller, less basic insertion highlighted by a grey background. Blue background represents that *Toxoplasma gondii* and many species of the *Plasmodium* genus have a longer, lysine-rich insertion. N-term and C-term indicates that whether the C- or the N-terminal repeat unit is included in the alignment of the protein sequences. The numbers indicate the mean number of substitutions per site. Phylogenetic tree was created with iToL tool^52^ and was manually annotated. (**B**) The lysine-rich character of the motif is conserved amongst *Plasmodium* species and is present in at least one of the two repeat units of the *Plasmodial* CCT enzymes. Blue and red letters highlight the basic and acidic residues, respectively. Numbers in the parenthesis show the length and the net charge of the insertion of each CCT protein and repeat unit. Visual representation of the alignment was generated with ESpript 3.0 and manually annotated^45^.

*Plasmodium falciparum* CCT is in focus of research for a long time as a potential drug target due to its regulatory role in the phosphatidyl-choline de novo biosynthetic pathway. The duplication of catalytic and membrane-binding domains as well as the presence of *Plasmodium-*specific segments distinguishes *Pf*CCT from its metazoan CCT homologs and the functional relevance of its many traits are yet unexplored. While structural and biochemical studies delineated the catalytic mechanism of *Pf*CCT^12,43,44^, the precise regulatory mechanism including membrane binding and compartmentalization for this enzyme is less understood. Here, we used a mammalian cellular system based on the thermosensitive CCT-mutant CHO-MT58 to evaluate the function of the *Plasmodium-*specific structural elements of the *Pf*CCT protein. The mammalian pathogens of the *Plasmodium* genus contain a typically lysine-rich insertion in the catalytic domain of their CCT protein that is absent in higher eukaryotic organisms (Fig. 5A). Intriguingly, other protists of the *Apicomplexan* phylum also possess similar insertions, albeit with fewer basic residues within. Noteworthy, this insertion is conserved in both repeat units of all *Plasmodial* CCTs, except the *P. berghei* CCT that contains an insertion that is less abundant in lysine residues in its C-terminal repeat unit and primarily contains non-charged asparagine residues (Fig. 5B). We investigated whether the positively charged cluster of lysine residues in *Pf*CCT would allow a potential nuclear localization signal role for this segment.

The full length *Pf*CCT protein displays a diffuse localization pattern in both schizont stage parasites^41^ and the studied CHO-MT58 cell line (present study, Fig. 3). The large molecular mass of the pseudo-heterodimer *Pf*CCT protein prevents its passive nuclear transport, thus the presence of a nuclear localization signal is required for nuclear targeting of this protein. Our confocal microscopy analysis shows that the lysine-rich loop has a nuclear localization signal function as the lysine-rich loop truncated constructs C2ΔK and C2M2ΔK were primarily found in the cytoplasm, in contrast to the full length *Pf*CCT and the C2M2 constructs (Fig. 3). A possible explanation on why this lysine-rich motif is present in many of the *Plasmodium* species is the AT-rich genome and the tendency to generate indels, possibly due to slippage of DNA polymerase over the AT-rich, low complexity genomic regions, resulting in a biased use of AT-rich codons e.g. lysine or asparagine^53,54^. Nevertheless, it is intriguing that a similar insertion is visible in the CCT protein of *Toxoplasma gondii,* a taxonomically close relative of *Plasmodium* species, despite its 52% GC content genome^55^. Here, DNA polymerase slippage -driven emergence of the insert is less likely, which in turn raises the possibility for the functional relevance of this insert in parasites. Notably, a similar insertion with importin-dependent NLS function was also reported previously in the *Plasmodium falciparum* trimethyl-guanosine synthase enzyme^56^. Additionally, low complexity, Lys-rich repetitive regions of *Plasmodial* proteins are reported to modulate protein targeting into the periphery of infected red blood cells^57^. The reason why the presence of a Lys-rich insertion is advantageous for the parasite is still unclear and requires further characterization.

Additionally, we investigated how the *Pf*CCT-expressing CHO-MT58 cells respond to PLC treatment that perturbs the phosphatidylcholine homeostasis. Our confocal microscopy analysis revealed a significant decrease in the nuclear fraction of both the full length *Pf*CCT and the construct C2M2 following PLC treatment. In comparison, the *Pf*CCT C2 construct that lacks the M domain displayed a decreased nuclear compartmentalization and the PLC treatment had no further effect on its localization. It was previously demonstrated that farnesol or oleate induced nuclear trafficking of exogenous CCT proteins in CHO-MT58 cells is dependent on amphipathic helix within M-domain^36^. Based on our results, the putative M domain of *Pf*CCT also influences nuclear-to-cytoplasmic localization though its precise function is still unclear as we found no evidence of translocation occurring in either M domain truncated construct. A notable difference in the function of M domain was highlighted previously as it was proposed that it consists of two, shorter membrane-induced amphipathic helices^24^. Furthermore, this disparity is linked to a mere sixfold activation in the presence of oleate^27^ that is in sharp contrast to the robust, up to 100-fold activation increase in case of rat CCT, accounting for a less fine-tuned *Pf*CCT regulatory mechanism^58^.

To evaluate the impact of the presence of the M domain on the exogenous *Pf*CCT activity in a cellular environment, we transiently transfected the CHO-MT58 cells with several truncated constructs and assessed their effect on cell survival at the restrictive 40 °C temperature. Our findings suggest that neither the lysine-rich insert in the catalytically important L5 loop, nor the putative regulatory M domain is essential to rescue the cells. Nonetheless, C1ΔK showed a slightly lower capability of rescuing the thermosensitive CHO-MT58 cells, though it was still significantly more potent than our inactive *Pf*CCT control. Moreover, we showed that *Hs*CCTα was also capable of rescuing the cells and further shows the possibility of a previously established concept to test *Pf*CCT-specific drugs by creating two, *Hs*CCT- and *Pf*CCT-expressing transgenic CHO-MT58 cell lines and use it as a cell-based test system for structure–function relation studies for drug development, targeting the PC biosynthetic pathway^42^.

We conclude that *Pf*CCT has adapted to provide a unique robustness in its functionality and has a key role in supporting the excessive need for PC biosynthesis during the intraerythrocytic development. Its unique membrane-binding domain as well as the Lys-rich insertion may contribute to the regulation of the parasite PC homeostasis and intracellular transport throughout the widely diverse developmental stages. As confocal imaging did not reveal any distinct membrane compartmentalization following PLC treatment, it would be of further interest to investigate whether the enzyme could serve the increased need for PC biosynthesis in absence of the membrane-binding induced regulatory mechanism throughout the intraerythrocytic life cycle of parasites. A notable limitation of our results, combined from several independent experiments, originates from the mammalian cell line-based model system. The true functional relevance of *Pf*CCT-specific structural elements has to be further assessed by in vivo experiments in parasites.

## Materials and methods

### Materials

Transient transfection was carried out with X-treme GENE HP transfection reagent (Merck KGaA, Darmstadt, Germany) according to the instructions of the manufacturer. Trypsin–EDTA (TE) solution and phosphate buffered saline (PBS) were used for general cell culture maintenance (Merck KGaA, Darmstadt, Germany). Plasmid pIRES-EGFP-puro was obtained from AddGene (#45567), provided by Michael McVoy (Virginia Commonwealth University). Enzymes *Phusion Hot Start Flex* DNA polymerase, *Nhe*I and *Xho*I restriction endonucleases and *Instant Sticky End Ligase Master Mix* are from New England Biolabs (Ipswich, MA, USA). Custom oligos were ordered from Sigma-Aldrich (Saint Louis, MO, USA). DNA purification kits for plasmid DNA and PCR product were obtained from Macherey–Nagel (Düren, Germany). Phospholipase C from *C. perfringens* (*C. welchii)* and every other reagent used here were obtained from Sigma-Aldrich (Saint Louis, MO, USA). Polyclonal anti-CCT was produced in rabbit after immunization with the second catalytic domain construct of *Pf*CCT_580–775, Δ720–737_ and was isolated in the Department of Immunology, Eötvös Lóránd University, Hungary.

### In silico analysis

Multiple sequence alignment was carried out by COBALT (NIH NCBI) with default parameters. To visually represent the alignment, ESpript 3.0 was used and manually annotated with the relevant secondary structure elements and domains. Homology model of *Pf*CCT including the lysine-rich loop was created with SwissModel, based on the crystal structure of the *Pf*CCT catalytic domain (PDB: 4ZCS)^44^ and was assessed using the *Structure Assessment* tool of SwissModel. Nuclear localization signal prediction was carried out with *cNLSmapper* for the entire protein with a cut-off score of 5.0. Helical representation of the different structural elements was made with HeliQuest using 3–11 helical structure^46^. Phylogenetic tree was generated with the Interactive Tree of Life (iTOL) tool^52^ after multiple sequence alignment of the different proteins using ClustalOmega.

### Cloning of *Pf*CCT constructs

The *Pf*CCT cDNA sequence (PlasmoDB: PF3D7_1316600) was cloned into pIRES-EGFP-puro plasmid. The cDNA sequence was then used to design primer pairs for the respective monomer and their truncated constructs. Constructs C1, C1M1, C2, C2M2, SV40-NLS C2M2 and their respective lysine-rich loop deleted (ΔK) versions (present in Fig. 1B) were amplified with the following primers (Table 1).

**Table 1:** Primers used to create the studied PfCCT constructs.

Primer name	Sequence (5′–3′)
C1-for	CGA**GCTAGCATGATCATCAAAGTGAACAGCG**
C1-rev	CGAT**CTCGAGCTATCCACGTTGCAGGCTACGTTCG**
C1M1-rev	CGAT**CTCGAGCTATTTTTTTTTTTTCAGTTCATCTTTCATGC**
C2-for	TCC**GCTAGCATGGTCGACCCGGATACCAATC**
SV40-NLS-C2 for	CAGC**GCTAGC**ATGGGAGCTTCACCCAAGAAGAAGAGAAAG GTG*GGTCGACCCGGATACCAATC*
C2-rev	GAC**CTCGAGTTATAGCTCATTCGGGTGGATG**
C2M2-rev	CACG**CTCGAGTCAGCTGCTGGTTTCATCATC**
C1ΔK for	CCGTACGCCAATAACCAGAAAGAAGATATCTACGCCTGGCTG
C1ΔK rev	CCGTACGCCAATAACCAGAAAGAAGATATCTACGCCTGGCTG
C2ΔK for	AACAATCAGAAAGAAGATATTTATGCTTGGCTGAAACG
C2ΔK rev	AATATCTTCTTTCTGATTGTTAGCATACGGGATGTC

Bold letters indicate the restriction sites of NheI and XhoI for the forward and reverse primers, respectively. Italic letters show the overlapping regions. Underscore shows the Simian Virus 40 NLS sequence that was used to generate the NLS-tagged construct (NLS C2M2). Lysine-rich loop deleted (ΔK) constructs were generated by QuikChange site-specific deletion^59^.

### Cell culture and rescue experiments

CHO-MT58 cells were kindly provided by Professor Dennis Vance (Department of Biochemistry, University of Alberta). Cells were cultured in an F12 Ham’s media supplemented with 10% foetal bovine serum (FBS) and 1% Penicillin–Streptomycin (Thermo Fisher Scientific, Waltham, MA, USA). CHO-MT58 cells were cultured at 37 °C and 40 °C in an incubator with a humidified, 5% CO_2_ atmosphere. Cells were prepared for rescue experiments seeding 100,000 cells in a 6-well-plate. At 80% confluence cells were transfected with 200 µl of transfection reagent mix containing 6 µl of X-treme GENE HP transfection reagent, 2 µg purified plasmid DNA in serum-free Opti-MEM medium. Media was always changed before the addition of the transfection mix. On the following day, culture was checked for transfection efficiency by live-cell imaging of the expressed green fluorescent protein (GFP) and if found proper, culture was split in half and incubated at 37 °C or 40 °C for an additional 3 days. Both cultures were then harvested. The supernatant was collected and mixed with the adherent cells resuspended by Trypsin–EDTA. To check the viability of cells, propidium-iodide (PI) was added in 1 µg/ml concentration. Flow cytometry was carried out with a *FACS Attune Acoustic Focusing Flow Cytometer*. After intact cells were gated based on Forward Scatter (FSC) and Side Scatter (SSC), GFP positivity and PI fluorescence were detected to determine the number of cells successfully transfected and the amount of dead cells. Rescue potential was calculated by the following formula:

$$Rescue \,  potential \, \left(\mathrm{\%}\right)=\frac{\frac{All \, cells-PI \,  stained \, cells }{All \,  cells}\,\, at\,\, 40^\circ C}{\frac{All \,  cells-PI \,  stained \,   cells }{All cells}\,\, at\,\, 37^\circ C}*100$$

### Immunofluorescent staining

50,000 CHO-MT58 cells were passed to a coverslip and were transiently transfected at 80% confluence with 50 µl of transfection reagent mix containing 1.5 µl of X-treme GENE HP transfection reagent and 0.5 µg purified plasmid DNA in Opti-MEM serum-free medium. After 24 h, PLC + cells were treated with 10 mU/ml phospholipase C for 3 h at 37 °C. Cells were then fixed with 4% paraformaldehyde for 10 min and permeabilized with 0.1% Triton X-100 solution. A blocking solution containing 3% bovine serum albumin (BSA) and 5% FBS in PBS was added for an hour. After the blocking, polyclonal primary antibody anti-CCT was added in 1:1000 ratio for an hour. Secondary antibody anti-rabbit Alexa Fluor 633 from goat was added in 1:1000 ratio after the primary antibody treatment for an additional hour. Finally, the cells were stained with 0.1 µg/ml 4′,6-diamidino-2-phenylindole dihydrochloride (DAPI) in PBS. After every step, the coverslips were washed 3 times with PBS. Every step was carried out at room temperature. The stained slides were embedded in *FluorSave* antifading agent to preserve fluorescence. Confocal imaging was carried out on a Zeiss LSM 710 microscope using a Plan-APOCHROMAT 40 × oil immersion objective.

### Colocalization analysis

8-bit images acquired by the confocal imaging were analysed in *Fiji*^60^ by the Coloc 2 plug-in. Threshold regression was carried out with the Costes method on images with determined regions of interest (ROI). Mander’s correlation coefficient was used to determine the nuclear overlap coefficient based on the DAPI and the corresponding anti-CCT signal using the following formula: $M=\frac{\sum_{i}{S1}_{i,coloc}}{\sum_{i}{S1}_{i}}$, where S1_i,coloc_ equals S1_i_ (intensity of the anti-CCT) signal if DAPI is above the determined threshold.
